# The importance of communication and involvement in decision-making: A study in Ireland exploring birth satisfaction using the Birth Satisfaction Scale-Revised (BSS-R)

**DOI:** 10.18332/ejm/162943

**Published:** 2023-06-19

**Authors:** Jean Doherty, Barbara Coughlan, Sophie Lynch, Lucille Sheehy, Caroline Hollins Martin, Colin Martin, Mary Brosnan, Martina Cronin, Theresa Barry, Ann Calnan, Sally Horton, Sharon Egan, Denise O'Brien

**Affiliations:** 1 National Maternity Hospital, Dublin, Ireland; 2 School of Nursing, Midwifery and Health Systems, University College Dublin, Dublin, Ireland; 3 School of Health and Social Care, Edinburgh Napier University, Edinburgh, United Kingdom; 4 Institute of Health and Wellbeing, University of Suffolk, Ipswich, United Kingdom

**Keywords:** birth satisfaction, maternity care, BSS-R, birth, Ireland

## Abstract

**INTRODUCTION:**

Evaluation in healthcare services has become a priority, globally^1^. The Government of Ireland has highlighted the importance of stakeholder engagement to identify the needs of women in the design and delivery of high-quality health services, driven by necessity rather than financial ability^2^. The Birth Satisfaction Scale-Revised (BSS-R), an internationally validated tool, and recommended for measuring childbirth satisfaction by the International Consortium for Health Outcomes Measurement (ICHOM)^3^; however, it has yet to be considered in the Irish context. The aim of the study was to explore birth satisfaction with a sample of new mothers in Ireland.

**METHODS:**

A mixed-methods study was conducted including a survey that involved collection of data from the BSS-R 10-item questionnaire from 307 mothers over an 8-week period in 2019, in one urban maternity hospital in Ireland. Quantitative and qualitative data were collected. Qualitative data from the free-text comments of the survey questions were analyzed using content analysis.

**RESULTS:**

Overall, women reported positive relationships with their care providers and were satisfied with the communication and support they received, as well as high levels of control and choice. Postnatal care, however, was highlighted as being less satisfactory with staffing levels described as inadequate.

**CONCLUSIONS:**

Understanding women’s birth experiences and what is important to them could facilitate midwives and other health professionals to improve the quality of their care and develop guidelines and policies that focus on women and their families’ needs. The vast majority of women rated their birthing experience as extremely positive. The main elements of care that contributed to a positive birthing experience for women were quality relationships with clinicians, choice and control, and emotional safety.

## INTRODUCTION

Historically, measures of maternity care quality tended to focus on clinical outcomes relating to cesarean section rates and morbidity and mortality rates. This focus on measuring technical aspects of maternity care in Europe was questioned by Escuriet et al.^4^ who called for a need to measure other factors that contribute to well-being, including satisfaction, communication and overall support during birth. The woman’s voice is essential to evaluate and inform the care given, and inform and guide quality improvement initiatives at a local and national level. One way in which quality of care from patients’ perspectives has been assessed internationally is through the development and application of satisfaction measures^5^.

‘Birth satisfaction’ is a term that encompasses a woman’s evaluation of her birth experience. Perception of the childbirth experience is highly personalized, and women’s views vary regarding what constitutes a positive and satisfying experience^6^. Indeed, the meaning of birth satisfaction is diverse and may take many forms^7^. It is defined as an individual life event incorporating interrelated subjective psychological and physiological processes, influenced by social, environmental, organizational and policy contexts^8^. According to Srivastav et al.^9^, determinants of maternal satisfaction cover all dimensions of care across structure, process, and outcome. Other important individual constructs of birth satisfaction include being respected, in control and listened to^10^. Satisfaction is, therefore, a construct that is complex and may change over time. It is known for some time^11^ that satisfaction is multidimensional – women may be satisfied with some aspects of their experiences and dissatisfied with others, and positive and negative feelings can coexist. Woman’s dissatisfaction with her childbirth experience may have immediate and long-term effects such as her health and her relationship with her infant, postpartum depression, post-traumatic stress disorder, fear of childbirth, negative feelings towards her infant, poor adaptation to the mothering role and breastfeeding problems^11-13^.

It is widely recognized that satisfaction is difficult to measure. In addition, the plethora of tools developed to evaluate dimensions of the experience is a further demonstration of its complexity^14^. Because of the complexities associated with birth satisfaction, it is understandably complex and difficult to measure accurately. Additionally, women are much less likely to be critical of their care when interviewed or asked to complete a questionnaire by a health professional directly involved in their care. Therefore, issues such as timing of data collection and having a gatekeeper need to be taken into consideration, as well as the choice of instrument. The importance of evaluation in healthcare is widely acknowledged by policymakers in Ireland and worldwide^2,15^. In terms of evaluating women’s childbirth experiences, this importance cannot be underestimated, due to the psychological and social impact of negative birthing experiences on a woman and her family^16,17^. Advancing an understanding of women’s experiences of childbirth has both practical and political relevance in current healthcare systems to guide practice, policy formulation, and research. However, little is known about women’s experiences of childbirth in Ireland. Indeed, very few evaluations have taken place that have specifically reported women’s satisfaction with their childbirth experiences. The aim of this study is to address this gap and measure birth satisfaction in the Irish context.

## METHODS

### Study aim

The aim of this study is to explore birth satisfaction in a sample of new mothers in Ireland. Quantitative and qualitative information was collected in a mixed-methods study design. This article presents the qualitative findings. It is planned to present the quantitative findings in a separate publication.

### Setting

The setting for this study is a stand-alone maternity hospital – a national referral center with an average of 8.5 thousand births per year at the time of data collection (8434 in 2018 and 8700 in 2019^18^. Women attending the hospital have the choice of attending obstetric-led or midwifery-led care, although most women attending the unit receive the active management of labor^18^. All women, regardless of whether they choose private, semi-private, public, or midwifery-led care, are cared for one-to-one, by a midwife, for the duration of their labor and birth. If the women have chosen private obstetric care or if any complications arise during their labor or birth, an obstetrician will also be present for and in some cases facilitate the birth (e.g. instrumental and cesarean births).

### The Birth Satisfaction Scale-Revised

The BSS-R is a 10-item self-report scale reduced from the original 30-item BSS^19^. The BSS-R assesses women’s perceptions of birth to determine women’s satisfaction with the childbearing experience^7,19^. The BSS-R 10 items are assessed on a 5-point Likert scale. The main advantage of using the BSS-R over the original 30-item BSS is the reduction in time it takes a postnatal mother to complete, without a reduction in validity. To provide a more in-depth overview of women’s perspectives and views, opportunities to provide free-text comments were offered after every item on the scale and a section at the bottom for women to provide further thoughts/comments. The ten items included in the BSS-R and the sub-themes in which they are categorized are presented in Table 1. As of mid-2020, the BSS-R has been used in over 39 countries to evaluate women’s childbirth experiences. The BSS-R was the chosen tool for this study as it is the recommended tool for measuring childbirth satisfaction by the International Consortium for Health Outcomes Measurement (ICHOM)^3^. To our knowledge, this is the first study to use/validate the scale in Ireland.

**Table 1:** BSS-R individual items and sub-scale categories

*No.*	*Items*	*Sub-scale categories*
1	I came through childbirth virtually unscathed	Stress
2	I thought my labor was excessively long	Stress
7	I found giving birth a distressing experience	Stress
9	I was not distressed at all during labor	Stress
3	The delivery room staff encouraged me to make decisions about how I wanted my birth to progress	Quality of care
5	I felt well supported by staff during my labor and birth	Quality of care
6	The staff communicated well with me during labor	Quality of care
10	The delivery room was clean and hygienic	Quality of care
4	I felt very anxious during my labor and birth	Women’s attributes
8	I felt out of control during my birth experience	Women’s attributes

### Data collection

Data collection took place over an 8-week period during summer 2019. All women were given an information leaflet prior to leaving the labor and birthing suite, informing the women of the study and that they would be invited to complete a questionnaire in the coming days. They were informed that completion was entirely voluntary. A member of the team, not involved in the women’s care during their labor and birth then distributed the questionnaire to women following birth on the postnatal wards and collected them prior to discharge from hospital/community care. Verbal information was provided again at this point and women were given the opportunity to ask questions about the research, prior to completion of the questionnaire. Written informed consent was received. In total 307 questionnaires were distributed and a 100% response rate was achieved. Women who suffered a stillbirth or perinatal death were excluded from the study. A total of 200 women included free-text comments. This article reports the findings from the rich data obtained from the comments and written responses to the open questions offered on the 10-item scale.

### Data analysis

A deductive, data-driven content analysis approach was adopted to interpret the data^20^. Martin et al.^21^ who developed the BSS-R recommend that the qualitative findings derived from the free-text comments be analyzed thematically under each of the ten BSS-R items. A coding frame was developed and was generated in a concept- and data-driven manner. The approach recommended by Mayring^22^ was adhered to while generating the coding frame to maximize validity and reliability. The method selected to code the data was open coding. Once all the data were coded, the software package NVIVO 9 visually displayed the codes developed. Similar codes were grouped as a theme, and the relationships between themes were then sorted into categories. This process was conducted several times to ensure that: 1) all the relevant data were coded; and 2) there was no duplication of themes presenting the data. After initial data analysis, data were sent to a second researcher who familiarized herself with the data and the themes developed. Both researchers then reviewed and deliberated on the themes, refining where necessary.

## RESULTS

In total, 307 respondents (postnatal mothers) completed the BSS-R survey questionnaire. Of those women, 200 included free-text comments to at least one of the items and/or the free-text comment section at the end of the survey, so rich descriptive qualitative data were obtained. A total of 614 comments were offered. The demographic characteristics of the women who participated are presented in Table 2.

**Table 2:** Characteristics of participants who completed the BSS-R at the Urban Irish Maternity Hospital, 2019 (N=307)

*Characteristics*	*Total (N=307) n (%)*	*Primigravida (N=127) n (%)*	*Multigravida (N=180) n (%)*
**Birthing unit**			
Delivery suite	200 (65.1)	79 (62.2)	121 (67.2)
Theatre	104 (33.9)	47 (37)	57 (31.7)
Other	2 (0.7)	0 (0)	2 (1.1)
Unknown	1 (0.3)	1 (0.8)	0 (05)
**Type of childbirth**			
Normal vaginal	165 (53.7)	51 (40.2)	114 (63.4)
Ventouse	21 (6.8)	18 (14.2)	3 (1.7)
Forceps	6 (2)	5 (3.9)	1 (0.6)
Ventouse and forceps	2 (0.7)	2 (1.6)	0 (0)
Pre-organized CS	66 (21.5)	17 (13.4)	49 (27.2)
Emergency CS	44 (14.3)	32 (25.2)	12 (6.7)
Other/unknown	3 (1.0)	2 (1.6)	1 (0.6)
**Pain relief** (except pre-organized CS)	**Total (N=241)**	**Primigravida (N=110)**	**Multigravida (N=131)**
Epidural	105 (43.5)	56 (50.9)	49 (37.4)
Entonox	50 (20.7)	10 (9.1)	40 (30.5)
Other pharmacological	8 (3.4)	3 (2.7)	5 (3.8)
Non-pharmacological	4 (1.7)	1 (0.9)	3 (2.3)
None	27 (11.2)	4 (3.6)	23 (17.6)
Other	29 (12.0)	23 (20.9)	6 (4.6)
Unknown	18 (7.5)	13 (1.8)	5 (3.8)
**Labor length** (hours)	**Total (N=307)**		
<1	11 (3.6)		
1–3	61 (19.9)		
4–6	66 (21.5)		
7–9	32 (10.4)		
10–12	23 (7.5)		
13–15	5 (1.6)		
16–18	4 (1.3)		
19–21	6 (2.0)		
22–24	1 (0.3)		
>24	16 (5.2)		
Unknown	6 (2.0)		
Not applicable	76 (24.8)		

CS: cesarean section. Other pharmacological: paracetamol, pethidine. Non-pharmacological: hypnobirthing. Other: morphine, spinal, anesthesia.

### Themes

Our data from the 200 women who wrote free-text comments were analyzed using 7 of the above 10 sub-scales/themes, as participants did not comment on the cleanliness or hygiene standards in the delivery room, or to the comment: ‘I came through childbirth virtually unscathed’. Additionally, none of respondents suggested in their free-text comments that their labor was, or was not, too long. For that reason, there was not enough data on these three questions to develop a theme.

### Quality of care: I felt well supported by staff during my labor and birth

In terms of the sub-scale quality of care, a central theme identified from women’s responses was the importance of support to women’s overall perceptions of their birth experience. Women felt well supported and feeling supported was linked with multiple positive outcomes. Much of the comments related to support referred to the personal attributes of the staff, particularly their kindness and caring nature:

*‘Very professional one-on-one service … exceedingly kind, caring, knowledgeable midwives.’*

*‘Delivery staff were amazing, kind, caring, professional, made us feel safe and supported.’*

Indeed, the most common word used throughout the responses was ‘support’ – the amount of support participants received from professionals was a consistent theme throughout the findings of the study. Participants offered in-depth descriptions of the support and help they received and how it impacted on their overall experience, as indicated in the following statements:

*‘Overall, my experience here was very good … The midwives and other staff were highly professional, supportive and kind, and I feel really privileged to have been here for this precious time’.*

*‘Honestly my experience was amazing. It is such a vulnerable time and having amazing midwives helped me so much, I will forever be grateful.’*

### Quality of care: The staff communicated well with me during labor

A consistent finding was the importance of communication as a marker of quality of care. The most important aspects of professional support were communication, information, involvement in decision-making and giving women the freedom to express their feelings during labor, as indicated in the following statement:

*‘I’m so impressed with the level of communication at such a stressful time.’*

Despite most comments being extremely positive in terms of the care women received, a recurrent theme was that women felt the hospital was ‘short staffed’ and that maternity care professionals did not get the recognition they deserved based on their working conditions and the high-quality care they provide, as indicated in the following statement:

*‘The staff are run off their feet. My recovery was tough for this reason. It’s a disgrace … Staff are so nice but they did not have a second and this wasn’t great for recovery.’*

*‘Community midwives are fantastic, support post-delivery not the same, midwives very busy, not same level of support.’*

In particular, women described how midwives were overworked postnatally, stating that:

*‘…nurses have too many patients to look after therefore some women may get overlooked.’*

Other such statements are presented below with one mother stating that:

*‘…postnatal care should be given more attention by healthcare organizations.’*

*‘Lack of staff in the postnatal left care for upwards of 30 mothers, and their newborn infants [while I was present] in the hands if three staff overnight and an insufficient number of daytime staff which in turn lead to a level of care verging on neglectful.’*

**Figure 1:**
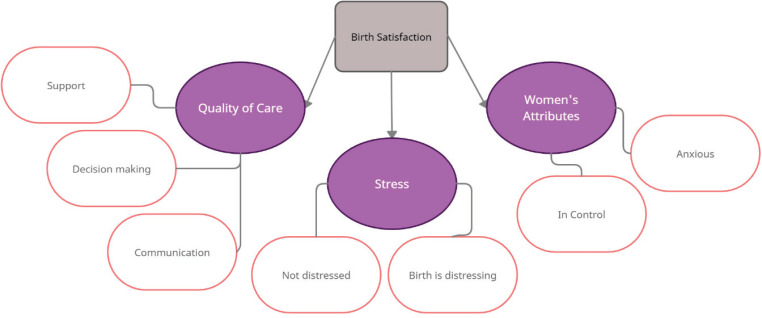
Birth satisfaction themes from BSS-R, using content analysis

### Quality of care: The delivery room staff encouraged me to make decisions about how I wanted my birth to progress

Assessments of quality of care around involvement in decision-making were incredibly positive. Women felt they were encouraged to make decisions and felt they were kept informed throughout their birth experiences. Participants suggested that the information they received contributed to their positive experiences. This was because they attributed making and participating in choices to a positive effect on their birth experience, as indicated in the following statement:

*‘The midwife in the delivery suite was fantastic from start to finish. She explained what was happening at every point in the process and this really helped, you know she involved us in all the stages, and this really helped, overall, a wonderful experience.’*

Some participants described taking personal responsibility in the pregnancy and for their own birthing processes and this was seen to have helped participants and their partners develop the understanding required to make informed decisions, an issue considered especially important if they were to feel in personal control of their birth processes:

*‘They were great at giving me the information so we could decide how best to proceed.’*

Factors that increased a woman’s chance of having a positive birth experience were reported as continuous information about labor progress, the opportunity to participate in decisions during labor, as well as a supportive and competent midwife and doctor, as indicated in the following statement:

*‘Due to low platelets and damage from a previous delivery, the delivery team took extremely good care of me. The management was second to none … calm and organized. I felt safe and well looked after given the complications.’*

Equally apparent was that participants felt informed throughout their experiences. This meant they perceived themselves to have felt equipped to make their own decisions. Therefore, this study’s data did not identify lack of autonomy as an issue, as indicated in the following statement:

*‘My overall experience was positive. Everything was explained thoroughly, and I was allowed to make my own decisions. I wasn’t rushed.’*

Because they described being encouraged to make decisions and choices about their birth preferences, many of the participants described their experience as empowering, as indicated in the following statements:

*‘I found the experience empowering.’*

*‘I had a very empowering birth on my first, almost four years ago. Obviously, I brought that to this experience.’*

### Stress: I found giving birth a distressing experience

Assessments of satisfaction with care around stress revealed that women generally did not find labor a distressing experience. Instead, participants frequently described feeling safe. This was not just about physical safety – it was evident that emotional safety was equally if not more important to them. The findings revealed that feelings of ‘being safe’ were associated with a supportive environment that enabled women to manage their labor. If a woman feels safe and well taken care of during the birth process, the overall experience was reported as positive despite any serious complications, as indicated in the following statement:

*‘As a first-time mother I knew labor would not be a walk in the park, but I don’t think anything could ever prepare you. There are so many different possible scenarios, and the length can vary dramatically. For me having experienced knowledgeable midwives looking after me was essential. It made a potentially terrifying experience very manageable from start to finish, no matter what happened.’*

Although some participants acknowledged how stressful childbirth is, the reassurances they received from staff were highlighted as helping allay their concerns and anxieties. Much of these concerns were related to anxieties about the safety and wellbeing of their baby and inherently linked with this was their perceptions about the competence of the staff. Indeed, a recurring theme was participants expressed confidence in the capability of the staff and how this impacted the level of distress they felt, as indicated in the following statement:

*‘Being rushed for an emergency section could have been distressing but with so many competent staff explaining everything in such detail made it less distressing. I knew I was in safe hands.’*

### Stress: I was not distressed at all during labor

What became apparent was that staff were perceived to be knowledgeable, and the information and support provided were commented on as reassuring and contributed to the emotional safety participants experienced. A central theme was how staff reduced anxiety levels and this helped participants feel relaxed and calm, as indicated in the following statement:

*‘Staff are great and help you a lot and keep you calm and relaxed as much as they can.’*

This sense of calm that participants experienced was linked with the sense of control exuded by staff, as indicated in the following statement:

*‘One of the main things for me was that I never felt that the medical side of things was ever out of control … the midwives and doctors were always in control, which helped us stay so calm. When the decision came to have a c-section, it was made with appropriate speed and consideration, and there was no ambiguity at all, this all contributed to our overall comfort and sense of calm.’*

As indicated below, being both professional and caring were viewed as important to participants overall experience and sense of calm:

*‘The staff who I met with during my delivery were professional and caring. I felt well looked after and confident in their ability to give myself and my baby the best care.’*

### Women’s attributes: I felt very anxious during my labor and birth

Most women’s accounts did not reveal high anxiety levels; however, the responses did reveal the individual nature of anxiety which was reported across a continuum. One respondent suggesting *‘No reason to be anxious’* and others revealing the personal nature of anxiety, as indicated in the following statement:

*‘I have a naturally anxious disposition and was anxious about induction.’*

Equally participants considered that some degree of anxiety was normal during childbirth as indicated in the following statement:

*‘I had normal levels anxiousness[sic].’*

Embedded throughout participants responses was the important role that support from staff played in reducing their anxiety, as indicated in the following statement:

*‘During the various stages I remained calm by using hypnobirthing techniques, but also because I was well informed and had a lot of support from staff, because of this I never felt any reason to panic or feel anxious.’*

### Personal attributes: I felt out of control during my birth experience

In terms of personal attributes around control, when women reported that they trusted their midwife, it facilitated their sense of choice and control, as indicated in the following statement:

*‘This labor was completely different to my first … both were positive experiences but as this one was much quicker and I mostly labored at home, I felt in control and the midwives were of great support throughout. I found that just that building of trust really helped in labor, because I was just so comfortable.’*

The data revealed that getting an outcome or experience that women originally wanted or expected was not as important to the women as feeling that they were maintaining some sort of control and involvement in the proceedings. The women’s quest for information and control helped them to have a fulfilling and satisfying birth experience even in situations where the birth experience did not meet their expectations:

*‘Emergency c-section due to failed induction but knew this was a possibility I like to be in control of any situation. When I felt like I had no control I was quickly reassured.’*

Having supportive midwives provided the women with an advocate who could continue to represent their views and offer support during labor and birth. This was an important factor in enabling women to feel in control, as per the following statement:

*‘Couldn’t control by body’s natural responses to labor but was in control of things I could be.’*

## DISCUSSION

Giving birth is one of the most important events in a woman’s life and is a highly individual experience. In addition, birth experience may have short-term and longterm effects on the woman’s physical and psychological health and the relationship with her newborn^11,12^. However, little is known about women’s experiences of childbirth in Ireland; indeed, very few evaluations have taken place that have specifically reported women’s satisfaction with their childbirth experiences. Women in this study provided extremely positive feedback about their birth experience in the research site. A total of 200 out of the 307 participating women explored and documented the most important aspects of their birthing experience, and although they were asked questions related to ‘stress’, their ‘personal attributes. and ‘quality of care’, it appeared from the findings that quality of care was central to most of their comments. The most frequent comments from participants related to feeling supported and listened to. Therefore, the findings of our study reveal that feeling supported and listened to are important constructs of birth satisfaction.

Indeed, the support that women received from professionals was a significant and consistent theme throughout the findings of the study. A significant long-term contribution to a positive birth experience is created when midwives demonstrate caring behaviors and offer tangible support to women during childbirth^23,24^ and participants described these caring behaviors and support provision from the staff they encountered. Examining the factors that can contribute to positive birth experiences, Thompson and Downe^25^ suggest that it is vital that women are connected to their caregivers and to their process and experiences of childbirth. The findings of this study support this perspective in that the women attributed the quality of their relationship with their caregiver as a key feature of their positive experience. Being involved during the decision-making process was equally important to the women in this study. Ensuring the provision of informed decision-making during childbirth is a key aspect of quality midwifery care^26^. Maternal empowerment is improved when a woman and her midwife share information that enables the woman to take the lead in decision-making and is one of the vital elements of compassionate midwifery^27^. In addition, our findings reveal that feeling informed enables women to understand and subsequently have confidence and trust in the decisions and choices of maternity care professionals. This is important because this form of confidence and trust in relationships are stressed as necessary factors for a positive birth experience for women^28^. Perceptions of control during labor and birth are also important. Women in this study who perceived themselves as experiencing greater control during childbirth reported increased satisfaction with the birth experience. Central to this was the relationships participants developed. Indeed, the mother–midwife relationship is an essential component in a woman’s perception of a positive birthing experience^29^ and the women in this study spoke favorably about the trusting relationships they built with their care provider. Studies show that a trusting relationship between a woman and her midwife is important for the emotional aspects of their birth experiences^28,30^. This is supported by the findings of this study, as women who reported positive relationships reported how the trust enabled them to make decisions for themselves. Equally, the findings of this study add to the literature on the importance of advocacy, choice, control, and communication to improve women’s birthing experiences.

Consistent with other studies, this study found that women were less satisfied with postnatal care compared with other aspects of maternity care^31,32^. Women in this study did not define a break between intrapartum and postnatal care when describing care received at the research site. Postnatal care is referred to as the ‘Cinderella of childbirth’^32,33^. In the current study, the reasons for this were perceived to be linked to staffing shortages, which have been reported for several years in Ireland, in the broader literature^34^. Unfortunately, chronic excessive workload caused by staffing shortages has been cited as the primary cause of midwife burnout in Ireland, which reduces quality of care^35^. It must be mentioned that data collection occurred during the summer months, which is a time when many staff take annual leave. Nevertheless, improvement in midwifery staffing levels has been recommended by the Health Information and Quality Authority (HIQA)^36^. To truly provide women with a complete and satisfactory birthing experience, efforts to improve staffing levels in the maternity care community need to be intensified.

### Strengths and limitations

This is the first time the Birth Satisfaction Scale-Revised (BSS-R) has been used in the Irish setting, and good quality data were collected and analyzed to explore women’s birth experiences in Ireland. Furthermore, the research team was made up of clinicians and academics with extensive qualitative and quantitative research experience. The main limitation is that the study was completed in one maternity setting only, a stand-alone maternity unit, which provides obstetric-led and midwifery-led services (both in the unit and in women’s homes). As such, the findings may not be representative of all maternity settings nationwide. Additionally, no comparisons were made in birth satisfaction in terms of labor onset or choice of care package (private, public, midwife-led etc.).

## CONCLUSIONS

Understanding how to improve women’s birthing experience could facilitate midwives and other health professionals to improve the quality of their care and developing guidelines and policies that focus on women and their families’ needs. This study allowed women to provide feedback on their birthing experience, with the vast majority rating it extremely positive. The main elements of care that contributed to a positive birthing experience for women were quality relationships with clinicians, choice and control, and emotional safety. Most significantly, the feeling of being supported was described the most, in terms of perceived good quality care. Improved staffing levels are imperative if we are to provide an excellent standard of postnatal care.

## Data Availability

The data supporting this research are available from the authors on reasonable request.
